# Oral Health Knowledge, Attitudes, and Clinical Practices of Pediatricians and Pediatric Residents: A Cross-Sectional Study

**DOI:** 10.7759/cureus.50785

**Published:** 2023-12-19

**Authors:** Deema Farsi, Dania Alagili

**Affiliations:** 1 Department of Pediatric Dentistry, King Abdulaziz University, Jeddah, SAU; 2 Department of Dental Public Health, King Abdulaziz University, Jeddah, SAU

**Keywords:** pediatrician, dental caries, prevention, children, oral health

## Abstract

Background

Pediatricians are the first line in the provision of healthcare for children. They can make an important contribution to the oral health of their patients because they usually see children and parents early and frequently in life. This study aims to assess the pediatricians’ and pediatric residents’ oral health knowledge, attitudes, and practices with patients.

Methods

A structured English survey was developed based on previous surveys in the literature. It collected data on oral health knowledge, attitudes, and practices. Pediatricians were visited and invited to take the survey. A link to the survey was sent via email to all pediatric residents. An oral health practice score was created based on participants' responses to the practice questions, and a linear regression assessed its predictors. Frequencies of oral health knowledge, attitudes, and practices were presented and compared between both groups by the chi-square test.

Results

A total of 218 pediatricians and residents were surveyed. The oral health knowledge was low overall, but it was higher among pediatricians, 10.0±1.9, compared to the residents, 8.2±2.5 (P<0.001). The attitude ranged from 66.3% agreeing with the statement “Limited time with patients makes it difficult to integrate oral health into primary care practice” to 87.4% agreeing with the statement “Primary healthcare physicians should know their local dentists to facilitate dental referral and treatment.” Overall, pediatricians practice positive oral health behaviors more than pediatric residents, and higher knowledge levels predicted more positive oral health practices.

Conclusion

Despite acknowledging their important role in promoting oral health, pediatricians' and future pediatricians’ knowledge is poor, and their participation in oral health continues to be limited. The potential for the non-dental workforce to greatly improve children’s oral health is underexploited. The healthcare system should seek to integrate medical and dental practices better. Incorporating oral health into residency programs and providing continuous education courses are strongly encouraged.

## Introduction

Oral health is an integral part of general health, and holistic health cannot exist without oral health [1]. Dental caries is the most common chronic childhood disease [2], with as many as 560 million children worldwide having dental caries in their primary teeth [3]. Early childhood caries (ECC), a rampant form of dental caries that occurs in infants and children under six, constitutes a serious public health problem. The prevalence of ECC varies by country but has been reported to affect up to 70% of disadvantaged children [4]. ECC causes dental pain and infection, which can have negative effects on nutrition, speech, communication, and the ability to learn and thus impede the child’s normal growth and development [5,6]. Furthermore, untreated caries has been associated with poor oral health-related quality of life and sense of well-being and increases the risk of future emergency visits and/or hospitalization [5,7,8]. Poor dietary and oral hygiene practices are major causes of ECC. Other risk factors include low socioeconomic status, being part of a marginalized population, low birth weight, and the transfer of cariogenic bacteria from mother to child [4]. In Saudi Arabia, the prevalence of caries was estimated to be 80% among young schoolchildren [9], and the prevalence of ECC varied from 30-80% depending on the region of study [10].

For two decades, the American Academy of Pediatric Dentistry (AAPD) has been encouraging parents to take their children to their first dental visit by their first birthday [11]. The purpose of this early visit is not limited to assessing the child’s oral health. It also offers an excellent opportunity to give parents age-specific anticipatory guidance that includes information on oral hygiene, the infectivity of dental caries, risk factors for traumatic injuries, the effects of non-nutritive habits, and dietary counseling [12]. In 2006, the AAPD adopted the concept of the “dental home,” based on the “medical home” in medicine, to deliver comprehensive, continuously accessible, coordinated, and family-centered oral health care. Prevention achieved by establishing a dental home by one year of age can decrease the need for later dental treatment and reduce its financial burden [11]. This recommendation was also adopted by the American Academy of Pediatrics (AAP) [13,14]. Despite these efforts, only a minority of children see a dentist before the age of three, and it is usually done only after caries have already been established [15-22]. A survey of parents attending the dental clinic at the King Khalid University College of Dentistry in Abha, Saudi Arabia, regarding the age of their child at their first dental visit, found that 8% of children visited the dentist for the first time before the age of one year and 17% between the ages of three and six years. Toothache was the main (40%) reason for these visits [23].

Unlike dentists, pediatricians generally see children in their first week of life, followed by regular, periodic visits throughout the first three years of their lives for optimal care [24]. Almost all children see a physician for multiple medical well-baby visits in their first two years of life and are likely to see a physician as many as 13 times before they ever visit a dentist, especially since primary medical care is more accessible than dental care for many families [25-27]. In these routine check-up visits, primary care physicians administer immunizations, track growth and development, and manage and monitor any concerns the child’s parents may have [24]. They also educate parents and caregivers on proper nutrition practices and discuss developmental milestones, social behavior, and learning [24]. Physicians can also check the children’s oral health early in life to promote good oral health and prevent oral disease. They can screen children for early signs of dental caries, implement preventive measures, and refer children in need of dental care [28]. In fact, the AAP policy statement on oral health care for children recommends that pediatricians be knowledgeable about dental caries, its prevention, and interventions to restore oral health and maintain holistic health [29]. Nonetheless, a review of 42 studies from 19 countries found that most pediatricians’ oral health knowledge was generally poor [30].

In 2014, the AAP published a policy statement on maintaining and improving young children’s oral health, which emphasized the role of pediatricians in promoting oral health and included detailed preventive strategies that pediatricians could utilize, in addition to urging collaboration with dental care providers [31]. A 2011 study in Saudi Arabia, published before the release of the AAP policy statement, found pediatricians’ oral health knowledge, attitude, and practice to be unsatisfactory [31,32]. Another study of Saudi Arabian pediatricians published in 2019 reported discrepancies between their oral health knowledge (42.6%), attitude (86.1%), and practice (65.3%) [33]. In addition, a 2019 study of Saudi Arabian pediatricians and pediatric residents found that only 21.9% of them were familiar with the AAP oral health guidelines, and 8.3% followed these guidelines in their regular practice [33]. While these local studies assessed pediatricians’ knowledge, attitudes, and practices in terms of children’s oral health care, none have examined the predictors of pediatricians’ oral health practices.

Although the present body of literature covered the topic of oral health knowledge among pediatricians, based on our information, there was no study that compared two cohorts, pediatricians and pediatric residents, with a substantial difference in years of experience, which we believe is important to determine changes in trends in the acquisition and application of this knowledge. Furthermore, studies that examined predictors pertaining to practices were scarce. Moreover, it was essential to assess the knowledge and practices of a local population with a more rigorous methodology. Thus, the current study was carried out with the aim of assessing the oral health knowledge, attitude, and practices of pediatricians in comparison to those of pediatric residents and examining the impact of their knowledge and attitude on their oral health practices. We hypothesized that oral health knowledge is insufficient in the target population.

## Materials and methods

Ethical approval, study design, and population

This is the first part of a three-part cross-sectional study aiming to comprehensively assess where primary healthcare physicians and residents stand regarding oral health. It was conducted following the principles of the Declaration of Helsinki. Approval was obtained from the Research Ethics Committee, Faculty of Dentistry, King Abdulaziz University (KAU), Jeddah, Saudi Arabia (063-06-20), in addition to the approval of the Institutional Review Board of the Ministry of Health (MOH) (20-665E). The population of the current study included the 123 pediatricians practicing at MOH hospitals and primary healthcare centers (PHCs) in the city of Jeddah, Saudi Arabia. Although there are pediatricians practicing in other governmental and private hospitals and medical centers, considerable differences were not expected between them. Thus, one broad group was chosen for this study. It also included the 298 residents enrolled in the Saudi Board of Pediatrics program in Jeddah with the support of the Saudi Commission for Health Specialties (SCHS), which oversees the Saudi Board specialty programs.

Survey

After a comprehensive review of the existing literature, a comprehensive English self-administered survey was developed [32-37]. The survey contained 36 closed-ended questions that were divided into two parts. The first part collected general demographic data. The second part assessed the participants’ oral health background and was further divided into three domains: knowledge (n=13), attitudes (n=5), and practices (n=9). Five domains of oral health knowledge were tested: general dental knowledge, the medical-dental relationship, prevention of oral disease, caries manifestation and etiology, and tooth development.

The comprehensibility and reliability of the survey were examined by three experts at KAU who were not part of the study team: a professor in pediatric dentistry, an associate professor in dental public health, and an associate professor in pediatrics (Cronbach’s alpha = 0.85). Ten pediatricians and 10 residents not in the target population pilot-tested the survey to assess its content and face validity. Once a final version of the survey was attained, it was digitized on SurveyMonkey's online survey portal (San Mateo, CA). We made all the questions in the online survey mandatory for the participants to answer to ensure there was no missing data in the study. The reported average time needed to complete the survey was seven minutes.

The purpose of the study was stated on the cover page of the online survey, along with the contact information of the principal investigator. A clear statement instructing them to complete it only once was posted in the email and cover letter to prevent participants from responding more than once. The first question was used to exclude ineligible participants. An informed consent to enroll in the study was obtained from each participant before participation. Participants’ names and contact information were not collected to ensure privacy and confidentiality, and participation was voluntary. To ensure the validity of the responses, an attention-testing question was added halfway through the survey with the following prompt: “We appreciate your attention. Please choose the word dentist from the list below.” Only the participants who chose “dentist” were included in the study.

Setting and participants

Recruitment was carried out differently for each population.

Pediatricians

In the city of Jeddah, there are 46 PHCs affiliated with five tertiary hospitals. Hospital and center directors were contacted for permission to visit them. A recently graduated general physician was hired and trained by the first author as a data collector. The aim of the study was explained to him, and he was given an iPad to collect responses on. He was instructed to meet with the pediatricians in person whenever possible to brief them on the study, and he asked them to take the survey on the iPad independently if they agreed to participate. When this was not possible, he was instructed to share the survey link with the pediatricians and encourage them to participate on their own.

Pediatric Residents

The SCHS was contacted, and the aim of the study was explained to them. They collaborated with the study by emailing the survey link to all residents registered with the Saudi Board Program in Pediatrics who were training in the city of Jeddah. Additionally, they sent out three reminder emails at two-week intervals.

Responses were collected over five months (September 2021 to January 2022), after which data were retrieved from the online survey software for analysis.

Study variables

The dependent variable, the oral health practices of pediatricians, was assessed by summing the scores of each oral health practice statement. Each statement was scored as follows: “never” = 0, “sometimes” = 1, “most of the time” = 2, and “all the time” = 3. As an exception, for the statement “I give patients sugary treats as a reward for good behavior,” the following scoring scheme was followed: “all the time” = 0, “most of the time” = 1, “sometimes” = 2, and “never” = 3. The scores ranged from zero to 27, with lower scores indicating lower levels of oral health practices.

The main independent variables were pediatricians’ oral health knowledge and attitude. The total oral health knowledge score was calculated by summing the scores for the responses to the oral health knowledge questions. Correct answers were scored 1, while incorrect or “don’t know” answers were given 0. The knowledge scores ranged from 0 to 13, with higher scores indicating better knowledge. A total attitude score was also computed by summing the scores for the attitude statements. The responses to each attitude statement were scored as follows: “strongly disagree” = 0, “disagree” = 1, “neutral” = 2, “agree” = 3, and “strongly agree” = 4. The attitude scores ranged from zero to 20, with lower scores indicating a more negative oral health attitude, discriminating between oral health and general health (e.g., the belief that oral health promotion is not the physician's job).

Other independent variables included the presence of a dental clinic in the hospital, having a system for referral to the dental clinic, and having a linked medical-dental electronic patient record. Collected demographic information included status (pediatrician, pediatric resident), gender (male, female), and nationality (Saudi, non-Saudi). Job ranking was only recorded for pediatricians (specialists, consultants, etc.).

Statistical methods

Frequencies and percentages were calculated for categorical variables such as patient demographics, clinic characteristics, knowledge, attitude, and practice statements. Means and standard deviations for knowledge, attitude, and practice scores were calculated. The internal consistency of the attitude and practice questions was calculated using Cronbach’s alpha. Pediatricians’ and residents’ responses to knowledge, attitude, and practice questions were compared using a chi-square test, while knowledge and attitude scores were compared using a two-sample t-test. The predictors of oral health practice were assessed using linear regression; the assessed predictors were status (pediatrician, resident), gender, nationality, presence of a dental clinic in the hospital/center, system for referral to the dental clinic, linkage of patients’ medical records to their dental records, knowledge score, and attitude score. All variables that were assessed in the univariate model were entered into the multivariate model, except for “referral to the dental clinic,” due to a significant correlation between it and “presence of dental clinic in the hospital” and between it and “linkage of patients’ medical records to their dental records.” P-values of 0.05 indicated statistical significance. Statistical analyses were conducted using Stata 12.1 (StataCorp LP, College Station, Texas, USA).

## Results

**Figure 1:**
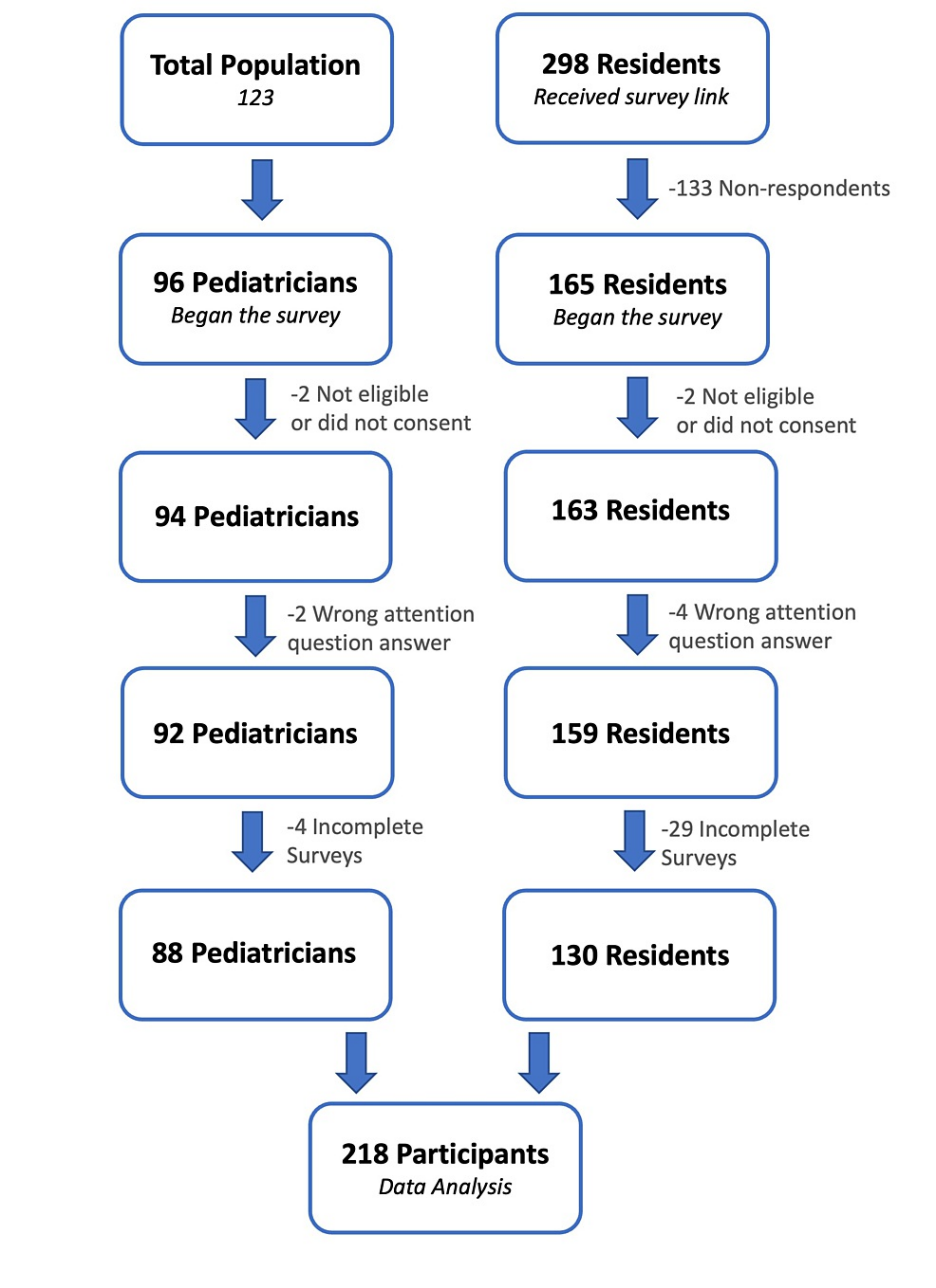
Flowchart of the participants

The survey was taken by 96 pediatricians, of whom eight responses were excluded. The survey was distributed to 298 pediatric residents, of whom 165 responded, yielding a response rate of 55.3%. However, only 130 pediatric residents were included in the analysis. Incomplete surveys, those with missing data, and those with a wrong answer to the attention question were excluded. Figure 1 is a flowchart of the participants. The sociodemographic characteristics of the participants are shown in Table 1.

**Table 1:** Sociodemographic and clinical characteristics of the study participants

Variable	Pediatricians N (%) (n= 88)	Pediatric residents N (%) (n= 130)
Gender		
Male	35 (39.8)	54 (41.5)
Female	53 (60.2)	76 (58.5)
Nationality		
Saudi	78 (88.6)	128 (98.5)
Non-Saudi	10 (11.4)	2 (1.5)
Rank		
Specialist	6 (6.8)	-
Consultant	71 (80.7)	-
Other	11 (12.5)	-
Presence of a dental clinic in the hospital		
No	5 (5.7)	35 (26.9)
Yes, located in the center	51 (58.0)	78 (60.0)
Yes, located outside the center but linked to it	32 (36.4)	17 (13.1)
Referral system to the dental clinic		
There is no referral system	4 (4.8)	16 (12.3)
Electronic	36 (43.4)	68 (52.3)
Paper-based	23 (27.7)	24 (18.5)
I don’t know	20 (24.1)	22 (16.9)
Patients medical record linkage to dental record		
Yes	40 (48.2)	61 (46.9)
No	25 (30.1)	34 (26.2)
I don’t know	18 (21.7)	35 (26.9)

Table 2 presents the participants’ responses to the 13 knowledge questions. The majority of the patients knew that teeth should be brushed at least twice a day (95%), while only one-third of the participants knew that bacteria that cause dental caries can be transmitted from mother to infant via saliva (30.7%). Only half of the respondents recognized that adding 0.7-1.2 PPM fluoride to drinking water helps prevent dental caries (55.5%). Overall, the knowledge of the participants was low, with a mean (SD) knowledge score of 8.9 +/- 2.4 out of 13, which corresponds to a score of 68.5%. Scores were higher among pediatricians, at 10.0 +/- 1.9, compared to the residents, at 8.2 +/- 2.5 (P<0.001).

**Table 2:** Oral health knowledge of the study participants * chi-square test, # two-sample t-test

Knowledge statement	All participants N (%) (n=218)	Pediatricians N (%) (n= 88)	Pediatric residents N (%) (n=130)	P-value
	Correct answers			
Children with no dental caries still have to see a dentist regularly	197 (90.4)	83 (94.3)	114 (87.7)	0.104*
The main causative organism of dental caries and gum diseases is dental plaque (bacteria)	140 (64.2)	68 (77.3)	72 (55.4)	0.001*
Oral infections can cause potentially life-threatening infections	200 (91.7)	83 (94.3)	117 (90.0)	0.256*
Malnutrition in pregnant women can cause defects in the baby's teeth	167 (76.6)	76 (86.4)	91 (70.0)	0.005*
Bacteria that cause dental caries can be transmitted from mother to infant via saliva contact	67 (30.7)	33 (37.5)	34 (26.2)	0.075*
Dental caries is the most common chronic childhood disease	101 (46.3)	51 (58.0)	50 (38.5)	0.005*
Individuals with special healthcare needs are at a high risk for the development of dental caries	186 (85.3)	84 (95.5)	102 (78.5)	0.001*
The first primary (baby or milk) tooth begins to erupt at around six months of age	195 (89.5)	84 (95.5)	111 (85.4)	0.018*
The child should see a dentist for the 1st time by 12 months of age	106 (48.6)	49 (55.7)	57 (43.9)	0.086*
Fluoride added to drinking water prevents dental caries	121 (55.5)	56 (63.6)	65 (50.0)	0.047*
Fluoride varnish is used to prevent dental caries in children five years and younger	84 (38.5)	47 (53.4)	37 (28.5)	<0.001*
Teeth should be brushed at least twice a day	207 (95.0)	86 (97.7)	121 (93.1)	0.124*
Overnight feeding (breast or bottle with any fluid other than water) can promote/enhance dental caries	179 (82.1)	79 (89.8)	100 (76.9)	0.015*
Knowledge score, mean (SD)	8.9 (2.4)	10.0 (1.9)	8.2 (2.5)	<0.001^#^

The participants’ responses to the attitude statements are illustrated in Table 3. Agreements with the attitude statements ranged from 66.3% agreeing to the statement “Limited time with patients makes it difficult to integrate oral health into primary care practice” to 87.4% agreeing with the statement “Primary healthcare physicians should know their local dentists to facilitate dental referral and treatment.” More residents (92.0%) agreed with the latter statement compared to pediatricians (81.4%) (P=0.025). Overall, attitudes were better among the residents compared to the pediatricians; attitude scores were 15.5 +/- 3.2 and 13.6 +/- 5.5, respectively, with P=0.002. The Cronbach’s alpha for the “attitude” section was 0.9.

**Table 3:** Oral health attitude of the study participants * chi-square test, # two-sample t-test

Attitude statement	All participants N (%) (n=199)	Pediatricians N (%) (n=86)	Pediatric residents N (%) (n=113)	P-value
		Agree/strongly agree		
Oral exams should be performed at each medical check-up visit	152 (76.4)	63 (73.3)	89 (78.8)	0.365*
Limited time with patients makes it difficult to integrate oral health into primary care practice	132 (66.3)	52 (60.5)	80 (70.8)	0.127*
Primary healthcare physicians play a very important role in promoting oral health among their patients	164 (82.4)	66 (76.7)	98 (86.7)	0.067*
Lack of knowledge and training in oral health care and trained auxiliary staff makes it difficult to integrate oral health into primary care practice	154 (77.4)	62 (72.1)	92 (81.4)	0.119*
Primary healthcare physicians should know their local dentists to facilitate dental referral and treatment	174 (87.44)	70 (81.4)	104 (92.0)	0.025*
Attitude score, mean (SD)	14.7 (4.4)	13.6 (5.5)	15.5 (3.2)	0.002^#^

**Figure 2:**
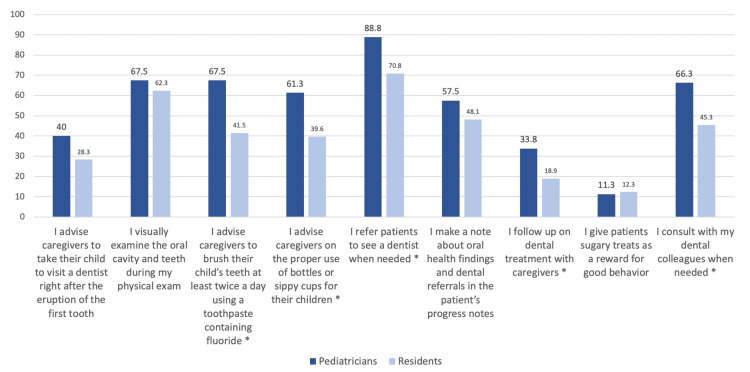
Distribution of oral health practices among study participants; responses of “all the time” and “most of the time” combined * indicates significantly different percentages between both groups at 0.05 level (chi-square test)

Figure 2 illustrates the distribution of pediatricians and residents performing oral health practices “all of the time” or “most of the time.” Overall, pediatricians practiced positive oral health behaviors more often than pediatric residents did. The most-performed oral health practice was referring patients to a dentist when needed; this was higher among the pediatricians (88.8%) compared to the pediatric residents (70.8%) (P=0.003). The Cronbach’s alpha for the “practice” section was 0.8.

The score assessment for predictors of good oral health practices is presented in Table 4. On average, pediatric residents reported a practice score that was 2.8 lower than that of pediatricians (95% CI: -4.2--1.4). The presence of a dental clinic and a referral system in the hospital, as well as the linkage of patients’ medical records to their dental records, were associated with higher practice scores compared to when these factors were absent. Knowledge score was also positively correlated with practice score; there was a mean 0.7 increase in practice score for each unit increase in knowledge score (95% CI: 0.1-1.0).

**Table 4:** Predictors of participants’ oral health practice (N=182) # Variables with missing values are not included in analyses ^ All variables that were assessed in the univariate model were entered in the multivariate model, except “referral to the dental clinic” * “Referral system to the dental clinic” was not entered in the multivariate model because of a significant correlation between it and “Presence of dental clinic in hospital” and between it and “Patients medical record linkage to dental record” (P=0.008) ‡ “Knowledge score” and “attitude score” were entered as continuous variables in the model

Variable	N^#^	Univariate regression	Multivariate regression^
Status			
Pediatrician	76	1.0	1.0
Resident	106	-2.8 (-4.2 - -1.4)	-1.1 (-2.6-0.4)
Gender			
Male	76	1.0	1.0
Female	106	-0.3 (-1.7-1.2)	-0.2 (-1.5-1.1)
Nationality			
Saudi	172	1.0	1.0
Non-Saudi	10	4.2 (1.1-7.3)	1.9 (-1.0-4.9)
Presence of dental clinic in hospital/center			
No	29	1.0	1.0
Yes	153	4.2 (2.3-6.1)	2.4 (0.3-4.4)
Referral system to the dental clinic*			
No	19	1.0	-
Yes	128	3.5 (1.1-5.8)	-
Don’t know	35	1.7 (-1.0-4.4)	-
Patients medical record linkage to dental record			
No	52	1.0	1.0
Yes	88	3.6 (2.0-5.2)	2.3 (0.7-4.0)
Don’t know	42	2.8 (0.1-4.7)	2.7 (0.9-4.5)
Knowledge score‡	182	0.7 (0.4-1.0)	0.5 (0.2-0.8)
Attitude score‡	182	-0.1 (-0.3-0.03)	-0.1 (-0.2-0.1)

## Discussion

This cross-sectional study is the first in a series of studies conducted on primary healthcare physicians to test their knowledge, attitudes, practices, competencies, and education related to oral health. The targets of the current study were pediatricians and pediatric residents, aiming to evaluate their oral health knowledge, attitudes, and practices with their pediatric patients. The findings showed that pediatricians were more engaged in positive oral health practices than residents and thus took a more active role in promoting oral health. Participants’ knowledge, the presence of a dental clinic in their hospital or center, and having their patients’ medical and dental records linked were among the predictors of their oral health practices.

We found that 95% of the participants knew that teeth should be brushed twice a day. However, less than 50% of participants agreed that children should see a dentist by 12 months of age, as recommended by the AAPD and AAP [11]. It seems that brushing your teeth twice a day is common knowledge. However, visiting a dentist for prevention early in life is a concept not yet well-established in the region. This result is slightly lower than the findings of a similar study in which 64% of pediatricians stated that children should see a dentist by age one year, but higher than the results of other studies in which 60% of pediatricians did not agree with this recommendation, and 33% of pediatricians did not know the recommended age for the first dental visit [35,38,39]. In a European survey, 43% of the surveyed pediatricians recommended a first dental visit for children older than three, while only 7% did for children under one [40]. There seems to be a universal lack of knowledge about the corrected age for the first dental visit, in accordance with the current findings.

Fluoride varnish application in the medical setting has been proven to be successful in reducing ECC occurrence and severity, especially with frequent early applications [41]. However, fewer than 40% of participants in the current study were cognizant of fluoride varnish. Knowledge about and skills in fluoride varnish application can only come with proper education and training, which seem deficient in this population. Knowledge about fluoride and its role in caries prevention was also found to be deficient in previous studies [35,42,43]. One study, however, found that most pediatricians and over 77% of family physicians appreciated the importance of topical fluoride for tooth caries prevention in children [44]. This may be because the study was conducted in the Niagara region of Ontario, Canada, where community water is not fluoridated, and healthcare providers have been regularly utilizing topical fluoride for caries prevention.

The lowest levels of knowledge were about dental caries manifestation and etiology; less than 40% of participants were aware that dental caries is the most prevalent childhood disease. Furthermore, only 30.7% knew that cariogenic bacteria can be transmitted to infants from caregivers via saliva. It was surprising to witness a lack of knowledge on the origin of cariogenic bacteria and the possible contagion of dental caries. Had knowledge in this area been better, it would be expected that primary care physicians advise pregnant women and new parents to engage in better oral hygiene measures and visit a dentist for any present carious lesions to decrease the levels of bacteria transmitted to their children. Our findings agree with previous studies in which only a few pediatricians and family physicians knew about ECC or understood that dental caries is an infectious and transmissible disease [34, 45]. Interestingly, a Lebanese study found that pediatricians with more than five years of experience were more likely to be knowledgeable about bacterial transmission than those with less experience [46]. In the domain of tooth development, 89.5% of participants were cognizant of the timing of the eruption of the first primary tooth. This is higher than the result of a similar study, in which only 74.3% of participating pediatricians were knowledgeable in this regard [38].

Overall, the participants’ knowledge was poor in the current study. Thus, we accept our hypothesis. It was not possible to differentiate between participants with good or bad knowledge. Nonetheless, the pediatricians generally had better oral health knowledge than the residents. We expected to find the opposite result since new medical graduates utilize evidence-based research and have easy access to information, whether through research articles or social media content [46,47]. Perhaps this assumption was incorrect due to residents’ under-utilization of resources or lack of interest in the current topic. A plausible explanation for the difference in knowledge between the two cohorts could be that the pediatricians acquired more oral health knowledge through practice. While some medical programs have started incorporating oral health topics into their curricula, this is not true for the region in this study [48,49]. In any case, the results of a 2018 US study suggested that there was little acquisition of oral health knowledge over the course of pediatric residency programs [27].

Both groups had a significant difference in attitudes; the pediatric residents reported more positive attitudes than the pediatricians. More than 82% of participants generally believed that they had an important role in promoting oral health. This acknowledgement is encouraging and lays a solid foundation for physicians to engage in oral health care when provided with knowledge and support. Almost all pediatricians in a 2009 study of fellows of the AAP agreed that they should examine children for dental caries [37]. In several studies, pediatricians have acknowledged their role in promoting oral health and integrating it into routine well-child visits [14,34,38,40,44,45,50,51]. Many pediatricians, however, have agreed that limited clinical time is a barrier to integrating oral health into their practices [37,38,44,51-53].

Our findings show that most (87.4%) participants agreed they should be aware of local dentists to facilitate dental referrals. In addition, most participants referred their patients to dentists when needed. These findings highlight the importance of establishing referral systems at institutional levels and encourage building professional communication channels with dental colleagues. Our finding agrees with Al Jameel et al.’s study, which reported that pediatricians referred children to dentists when they had obvious dental caries [33]. A study of Saudi Arabian medical students' oral health knowledge and practices found that 59% were making dental referrals based on caries risk and 25% on emergency needs [39]. These practices are not aligned with the AAP guidelines, which recommend that pediatricians refer children to a dentist by the child’s first birthday to establish a dental home [31]. To facilitate this, it is fundamental that physicians are aware of the local dentists and that interaction and communication channels between them are open. Furthermore, physicians need assurance that children have access to dental services to have confidence in their referrals. Pediatricians have noted that their local dentists do not accept children under three years old, therefore limiting their ability to refer [14]. Others did not refer young children to a dentist due to parental disapproval of this advice [54].

The AAP encourages pediatricians to examine children’s mouths during well-child visits and to recommend brushing teeth with fluoride toothpaste [55]. After dental referrals, pediatricians' second and third most common oral health practices in the current study examined patients’ mouths and advised parents to brush their children’s teeth. Oral examination is crucial in preventing dental caries and its progression in children, especially if the physician is cognizant of the manifestations of oral disease and can make dental referrals as needed. Fortunately, physicians provide advice about oral hygiene. However, the impact would have been greater had advice been given regarding feeding habits. Previous pediatricians’ reports on these practices vary by country and type of health facility, but most pediatricians advised parents to maintain good oral hygiene, recommended brushing with fluoridated toothpaste [33,56], and visually examined their pediatric patients’ oral cavities and teeth [34,35,44,45]. However, other studies showed less favorable findings [38,50]. Alshunaiber et al. found that most pediatricians and family physicians did not counsel parents regarding dental care [38]. In other studies, pediatricians inquired about children’s dental visits more often than they examined their teeth [57,58]. More than half of the participants were reluctant to examine children’s mouths because they felt it difficult to make dental referrals for these patients [37,58]. Furthermore, pediatricians have reported an inability to assess plaque and dental caries due to insufficient training [57,58].

In the current study, pediatricians had better oral health practices than residents. This supports the theory that positive practices may be gained and connections may be developed through experience. Pediatricians gave more oral hygiene instructions and performed more oral examinations than pediatric residents. A plausible reason could be that they directly oversee their clinical time and are thus able to incorporate oral health practices as needed. Perhaps they were more confident in their abilities to examine oral cavities. Additionally, pediatricians made more dental referrals and consulted dentists more often than residents. This could be attributed to developing more connections with dentists over the years, having more clinical time, and having more confidence in communicating with colleagues in other specialties. A study by Gereige et al. found that pediatric residents had poor confidence in identifying dental caries and assessing oral health risk factors [52]. In line with our findings, a study found that physicians who were confident in performing oral screenings and those who knew dentists who accepted referrals were more likely to make dental referrals [59].

We found that knowledge, rather than attitude, was a predictor of participants’ oral health practice. Similarly, a study found that pediatricians with more oral health knowledge were more likely to make dental referrals [60]. Another study found pediatricians' confidence in identifying dental caries was related to the frequency of their examination for oral diseases [57]. Moreover, another study observed the translation of knowledge into practice, as pediatricians with higher knowledge scores were more willing to perform caries prevention practices [61]. Another study observed no direct effect of oral health training on physicians’ oral health practices [62]. Beyond knowledge, the current study found that the presence of a dental clinic in the hospital/center and the linkage of patients’ medical records to their dental records positively influenced oral health practices. Surprisingly, being a non-Saudi Arabian pediatrician appeared to influence oral health practice as well positively. Attitudes did not appear to influence practices, suggesting that regardless of what participants believed to be their role, they engaged in oral health practices if they had the knowledge and a system that supported them.

The high rates of dental caries in Saudi Arabian children suggest an urgent need to address the unjust divide between children’s oral health and their general health. The present findings highlight missed opportunities in primary care settings for improving oral health for children. This is especially crucial in a population as young as Saudi Arabia, where 25% of people are younger than 15 [63]. Healthcare providers must consider new strategies to meet the needs of children and effectively tackle the caries epidemic. The potential for the non-dental medical workforce to improve children’s oral health is well documented [5,64]. Pediatricians usually see children earlier than dentists and have more frequent contact with their families. By improving their oral health literacy and incorporating oral health into the routines of well-childcare, they can help prevent oral disease, improving not only children’s oral health but their general health as well [65]. Studies have shown that a couple of hours of training is enough to help primary care physicians accurately diagnose caries and identify high-risk children who need dental referrals [41, 66-68]. Preventive measures initiated during the first years of life can significantly reduce the risk of developing dental caries and the need for more expensive restorative procedures later in life [43].

A limitation of the current study is its small sample size. Despite efforts to increase participation, the overall response rate was low, which is common in survey studies [45,52]. Moreover, there is a likely over-representation of participants interested in oral health, as they were probably the most willing to complete the survey. However, since physicians have more homogeneous characteristics than the general population, nonresponse bias is less likely to be a concern in this study [69]. Furthermore, data was collected differently: some participants were contacted in person while others received the survey link. To minimize any potential bias, participants filling out the survey in person did so privately and independently without the aid of the data collector. Finally, the reliance on personal reporting carries a risk of self-reporting bias. However, the anonymous nature of the responses can limit the effects of this potential bias.

Our findings demonstrate the persistence of a deficiency in oral health knowledge and implementation among pediatricians in Jeddah, which could be generalizable to other pediatricians in the country. From a public health point of view, it is unfortunately likely that dental caries rates remain high among children with limited access to dental care, and their general well-being is negatively affected. The burden of poor oral health on children, families, and the healthcare system is likely to persist unless the practice of marginalizing oral health is reversed. It is crucial that oral health be integrated into medical care and that the referral system between medical and dental care be facilitated. Breaking the dental caries cycle requires multifront efforts by the administrative ministry of health and the practicing medical and dental teams.

Our findings demonstrate the persistence of a deficiency in oral health knowledge and implementation among pediatricians in Jeddah and can be generalizable to all pediatricians in the country. Unfortunately, they project that the problem will continue unless the practice of marginalizing oral health is reversed. Unfortunately, it is likely that dental caries rates will remain high among children with limited access to dental care, and their general well-being will be negatively affected. The burden of poor oral health on families and the healthcare system is likely to persist.

The current findings will be presented to the medical school at KAU with a proposal to incorporate an oral health module into the undergraduate curriculum and to the SCHS to urge the incorporation of oral health into postgraduate medical residencies. Our goals are not short-term; we aim to establish connections with medical teams and introduce efficient and feasible oral health courses that are sustainable. We aspire to see this collaboration set a benchmark for regional medical schools, and thus, the effects will not only be local or even national but extend to geographically distant community-based pediatricians. Once pediatricians become well-versed in oral health and the implementation of oral examination and prevention becomes second nature to them, pragmatically, oral health among children will improve, caries prevalence will decline, the economic burden of caries from treatment costs and loss of productivity will be reduced, and the issue of limited dental care access will be understated.

Future research is needed to investigate the impact and feasibility of different oral health modules offered to medical students at the undergraduate and postgraduate levels. In addition, future research about the challenges and opportunities regarding improving children's oral health in a primary care setting is warranted.

## Conclusions

Despite acknowledging their important role in promoting oral health, pediatricians' and future pediatricians’ knowledge in this area is poor, and their participation in oral health promotion continues to be limited. They expressed support for but had less direct engagement in oral health activities in their practices and identified a lack of clinical time as a barrier. Achieving good oral health for all children requires the support of a wide range of healthcare professionals. Therefore, more oral-health-related training appears to be crucial for this population.

## Appendices

Abbreviations

EEC: early childhood caries

AAPD: American Academy of Pediatric Dentistry

AAP: American Academy of Pediatrics

KAU: King Abdulaziz University

MOH: Ministry of Health

PHC: Primary Healthcare Center

SCHS: Saudi Commission for Health Specialties

CA: California

SD: standard deviation

CI: confidence interval
